# Burnout and Quality of Life in Professionals Working in Nursing Homes: The Moderating Effect of Stereotypes

**DOI:** 10.3389/fpsyg.2022.772896

**Published:** 2022-03-04

**Authors:** Patricia López-Frutos, Gema Pérez-Rojo, Cristina Noriega, Cristina Velasco, Isabel Carretero, José Ángel Martínez-Huertas, Leyre Galarraga, Javier López

**Affiliations:** ^1^Department of Psychology and Pedagogy, School of Medicine, Universidad San Pablo-CEU, CEU Universities, Madrid, Spain; ^2^Department of Cognitive Psychology, Universidad Autónoma, Madrid, Spain

**Keywords:** ageism, stress, institutions, workplaces, quasi-experimental

## Abstract

**Objective:**

This study aimed to analyse how stereotypes towards older people moderate the relationship between burnout and quality of life (QoL) of professionals working in nursing homes.

**Method:**

A total of 312 professionals were asked to complete questionnaires of burnout Maslach Burnout Inventory quality of Life (QPL-35) and aging stereotypes (CENVE). The moderation effects were tested using linear regression models.

**Results:**

A negative association was observed between burnout and QoL. It was also found a statistically significant moderator effect of the total score of stereotypes and the health stereotypes, besides a marginally statistically significant moderator effect for motivational stereotypes. The moderator effects were different for people with low and high negative stereotypes. QoL was more affected under conditions of high burnout, in which people with more negative stereotypes tended to present higher QoL than people with lower negative stereotypes.

**Conclusion:**

Highly burned-out professionals in nursing homes may avoid their negative feelings projecting them to residents through stereotypes, as a way of coping with burnout and increasing their QoL. However, this mechanism is an “aggressive reaction” and may have negative effects for both the older adult and the professional. The comprehension of these variables is essential for developing adequate intervention programs.

## Introduction

Burnout is a syndrome that takes place after continuous exposure to workplace stressors (Maslach, 2009). It defined as a disorder that contains three dimensions: emotional (implies both physical and psychological exhaustion), depersonalization (decrease in motivation toward work), and a decrease in personal accomplishment (concept related to low self-esteem; Maslach and Jackson, 1981).

Workers in nursing homes face residents’ disrupted behaviors, diseases, pain, and suffering every day. The presence of these factors could increase the probability of suffering from burnout (El Haj et al., 2020; López et al., 2021). Other research also suggests that decreased job satisfaction, workplace issues, or tasks performed may be factors leading to burnout (Gutierrez-Martínez et al., 2021).

Burnout has been negatively associated with the quality of life of professionals working in nursing homes (Deví et al., 2016; Harrad and Sulla, 2018; Carrasco-Temiño, 2019).

According to the most accepted definition of Quality of Life, elaborated by the World Health Organization’s Quality of Life Assessment Group (WHOQOL, 1995), it is the expression of the person’s attitude toward life, within a cultural context and in line with their interests, purposes, or expectations. Burnout not only relates to professionals’ QoL but also to how the worker perceives other people (Maslach, 2009), including the older adults they work with. Regarding this, negative attitudes toward older adults have been associated with higher levels of burnout (Kane et al., 2018). Stereotypes are beliefs about different social groups, in which people are grouped by characteristics that are usually negative and ignoring the particularities of each person (Marx and Ko, 2019). Moreover, these negative stereotypes are the cognitive dimension of ageism that is considered a form of discrimination based on age (Rosell et al., 2020).

How the person perceives stressors moderates the impact of the stressful event and the consequences over their physical and psychological health (Lazarus and Folkman, 1984). In this sense, professionals’ beliefs and perceptions of the older adults they work with may moderate the impact of burnout on quality of life. However, this hypothesis has not been tested yet.

It is important to study nursing homes professionals’ burnout and its impact on their QoL not only for the need to protect and promote professionals’ safety, health, and wellbeing but also the older adults they are caring for. For example, found lower levels of good care in professionals with higher levels of depersonalization in nursing homes (López et al., 2021). Understanding the process of how burnout affects professionals’ QoL and how these variables are related to stereotypes toward older people will allow the development of interventions to promote the health of both workers and residents. This can be especially important during the current COVID-19 pandemic, which has provoked not only severe negative consequences on institutionalized older adults but also on staff working in long-term care facilities due to facing higher mortality rates and being exposed to stressful work and environmental factors (workload, lack of suitable protocols, equipment, and media pressure; Martínez-López et al., 2021).

Although the relationship between ageism and burnout has been extensively studied (Iecovich and Avivi, 2017), to our knowledge, the moderator role of negative stereotypes toward older adults over burnout and its impact on nursing home staff’s quality of life has not received attention yet. Taking this point into consideration, this study aimed to analyze the relationship between burnout and quality of life on professionals’ working in nursing homes and whether stereotypes toward older people moderate this relationship.

## Materials and Methods

### Participants

A total of 312 professional caregivers working in nursing homes in Spain participated in the study. A total of 86.90% were women and their average age was 40 years old (*SD* = 11.82; range = 19–70). A total of 14.8% of the sample did not perceive being overloaded at work, while 55.8% perceived something, 23.2% quite a lot, and 6.1% a lot of overload. Regarding job satisfaction, 2% showed low satisfaction, 43% medium, and 55% high levels.

### Instruments

Sociodemographic and professional variables: age, gender, work overload, and job satisfaction.

#### Maslach Burnout Inventory

This instrument was used to measure burnout (Maslach et al., 1996). It is composed of 22 items grouped in three factors: emotional exhaustion with nine items, depersonalization with five items, and personal accomplishment with eight items. In this study, we used the total score of the instrument. This version showed good reliability (Cronbach’s α = 0.87; McDonalds’ ω = 0.87).

#### Professional Quality of Life Questionnaire (QPL-35)

We used this scale to analyze quality of life (Martín et al., 2004). It includes 35 items and measures the balance between work demands and personal resources. This version showed good reliability (Cronbach’s α = 0.91; McDonalds’ ω = 0.92).

#### Negative Stereotypes Toward Aging Questionnaire (CENVE)

It is composed of 15 items (Mena et al., 2005). The factor structure presents three subscales with five items each: health (Cronbach’s α = 0.83; McDonalds’ ω = 0.83), motivation-social (Cronbach’s α = 0.66; McDonalds’ ω = 0.68), and character-personality (Cronbach’s α = 0.77; McDonalds’ ω = 0.78). This version showed good reliability (Cronbach’s α = 0.91; McDonalds’ ω = 0.90).

### Procedure

The study was approved by the University CEU San Pablo Ethics Committee. After that we contacted with different nursing homes (private and public; rural and urban ones) in Spain to inform their professionals about our study and ask them to collaborate. Participation was voluntary and confidentiality was guaranteed. Participants who agreed to participate in the study completed the informed consent and then completed a self-administered questionnaire which included sociodemographic information and the outcome variables: burnout, quality of life, and stereotypes. Participants completed the questionnaires in the nursing homes they were working and there was a trained psychologist in case they needed help. It took them 15 min approximately to complete the survey.

### Data Analysis

First, descriptive analyses were obtained to describe the sample of the study. Second, we standardized all the variables of the study to conduct the moderation analyses and to ease the interpretability of the results. Third, the moderation effects of stereotypes on the relationship between burnout and quality of life were tested using linear regression models for all the subscales of stereotypes. All the statistical analyses were performed in SPSS.24 software. Moderation analyses and graphical representations were performed using the *PROCESS macro v35* for SPSS software (Hayes, 2017; Hayes and Rockwood, 2017).

## Results

### Descriptive Analysis

Table 1 presents the descriptive analysis of the variables of the study. Descriptive analyses were performed using the original metric of the variables while the moderation effects were tested using standardized variables.

**Table 1:** Pearson correlation coefficients, descriptive analysis, and reliability of the variables.

**Variable**	**1**	**2**	**3**	**3.1**	**3.2**	**3.3**
1. Burnout	–	−0.29^**^	−0.01	−0.04	0.02	−0.01
2. Quality of life		–	−0.09	0.00	−0.15^*^	−0.11
3. Negative Stereotypes			–	0.90^**^	0.88^**^	0.91^**^
3.1. Health				–	0.69^**^	0.71^**^
3.2. Motivational-social					–	0.72^**^
3.3. Character-personality						–

*N* = 312.

* *p* < 0.05;

** *p* < 0.01.

### Moderator Effects of Negative Stereotypes

**Figure 1:**
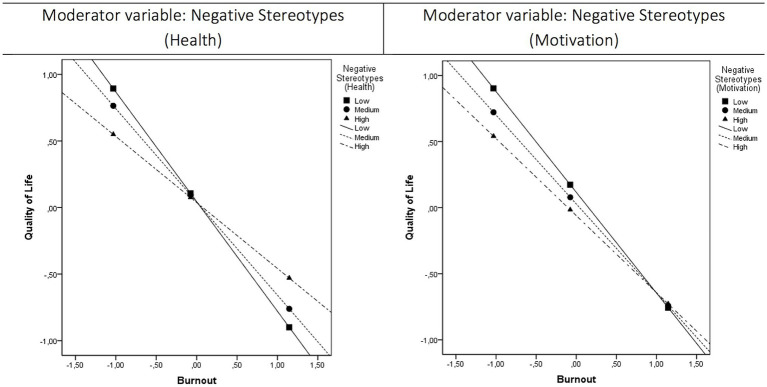
Graphical representation of the moderator effects of negative stereotypes (health) and negative stereotypes (motivation). Moderator effects were estimated using a continuous variable for negative stereotypes, but it was categorized in three groups (low, medium, and high negative stereotypes) using the PROCESS macro v35 for SPSS software to ease the interpretability of the moderator effect.

Table 2 presents the results of the moderator effects of stereotypes in the relationship between burnout and quality of life (moderator models M1–M4). All models presented a good prediction of quality of life (*R*^2^ ranged from 0.46 to 0.48). As expected, a negative relationship was found between burnout and quality of life. Negative stereotypes (general score) presented a statistically significant moderator effect (M1: *b* = 0.10, *SE* = 0.04, *t* = 2.18, *p* = 0.03). A similar moderator effect was found statistically significant for health stereotypes (M2: *b* = 0.14, *SE* = 0.04, *t* = 3.11, *p* < 0.001) and marginally statistically significant for motivational-social stereotypes (M3: *b* = 0.09, *SE* = 0.05, *t* = 1.79, *p* = 0.07). Figure 1 presents a graphical representation of the moderator effects of health stereotypes and motivational-social stereotypes. As the general score of negative stereotypes is a composite variable, it presented an average effect of both variables. It was found that the moderator effects differently influence the relationship between burnout and quality of life for people with low and high negative stereotypes. The slope of the participants with low negative stereotypes is larger than the one of the participants with high negative stereotypes. It means that the relationship between burnout and quality of life is higher for people with low negative stereotypes. Specifically, under conditions of low burnout, people with lower negative stereotypes tend to report more quality of life than people with higher negative stereotypes. On the contrary, under conditions of high burnout, people with more negative stereotypes tend to present more quality of life than people with lower negative stereotypes. This pattern of results can be observed for all the moderator effects but presenting different moderator effect sizes as the differences between groups tend to disappear in motivational-social stereotypes. On the opposite, no statistically significant moderator effect was found for character-personality stereotypes (M4: *b* = 0.06, *SE* = 0.05, *t* = 1.25, *p* = 0.21).

**Table 2:** Results of moderator models of negative stereotypes (M1–M4).

**Parameter**	**Estimate**	***SE***	**95%CI**	**Value of *t***	**Value of *p***	***R*** ^**2**^
(M1) Predictor = Burnout. Moderator = Negative stereotypes. Dependent variable = Quality of life.						
Intercept	0.03	0.05	−0.06—0.12	0.57	0.57	0.47
Burnout (DE)	−0.66	0.04	−0.75—−0.57	−0.1490	<0.001	
Negative Stereotypes (DE)	−0.03	0.05	−0.13—0.06	−0.71	0.48	
Negative Stereotypes (ME)	0.10	0.04	0.01—0.20	2.18	0.03	
(M2) Predictor = Burnout. Moderator = Health Stereotypes. Dependent variable = Quality of life.						
Intercept	0.04	0.04	−0.05—0.13	0.91	0.36	0.48
Burnout (DE)	−0.68	0.04	−0.76—−0.58	−15.37	<0.001	
Health Stereotypes (DE)	0.00	0.05	−0.09—0.09	−0.06	0.95	
Health Stereotypes (ME)	0.14	0.04	0.05—0.23	3.11	<0.001	
(M3) Predictor = Burnout 1. Moderator = Motivation Stereotypes. Dependent variable = Quality of life.						
Intercept	0.01	0.05	−0.07—0.10	0.31	0.76	0.47
Burnout (DE)	−0.66	0.04	−0.75—−0.57	−14.96	<0.001	
Motivation Stereotypes (DE)	−0.09	0.05	−0.18—0.01	−1.79	0.07	
Motivation Stereotypes (ME)	0.09	0.05	−0.18—0.01	1.79	0.07	
(M4) Predictor = Burnout 1. Moderator = Character stereotypes. Dependent variable = Quality of life.						
Intercept	0.02	0.05	−0.07—0.11	0.49	0.62	0.46
Burnout (DE)	−0.66	0.04	−0.74—−0.56	−14.69	<0.001	
Character Stereotypes (DE)	−0.04	0.05	−0.14—0.05	−0.93	0.35	
Character Stereotypes (ME)	0.06	0.05	−0.03—0.15	1.25	0.21	

M1–M4, Moderator models; SE, Standard error; DE, Direct effect, ME, Moderator effect.

## Discussion

Our results show that there is a relationship between the three variables studied. First, we found a negative association between burnout and quality of life. This is in the same line that other studies who stressed the idea of the affectation that this syndrome has on people’s quality of life (Grava et al., 2019).

Second, when analyzing the moderating effect of stereotypes on the relationship between burnout and quality of life based on Lazarus and Folkman (1984) model results showed a moderation effect of stereotypes global scale, health, and motivation subscales. In contrast, character stereotypes subscale did not moderate the relationship between professionals’ burnout and quality of life. Health and motivational scales refer to inaccurate attitudes in which older people are perceived as dependent and in decline, concepts that are associated with higher levels of workload (Bedoya et al., 2020), and being one of the main triggers of burnout (Zamora and Sánchez, 2008). In contrast, the character subscale refers to aspects related to personality, internal attitudes, which may not involve an extra workload for the professionals, and, therefore, may not moderate the relationship between professional’s burnout and their QoL.

We also observed a different impact of stereotypes depending on the levels of burnout. When there was a low level of burnout, the quality of life was less affected. In contrast, we have found that when there are high levels of burnout, stereotypes moderate in such a way that the more stereotypes the higher the quality of life, contrary to what theoretical approaches supports and our expectations.

These results may be associated with the use of the avoidance coping strategies, aggressive reaction (Londoño et al., 2006). By expressing their negative feelings against older people, professionals can perceive they increase their quality of life. Also, we can relate these results to the projection defense mechanism, which aims to protect the person who uses, by attributing the negative traits to others and, in this case, to the older adults. As a result, professionals may perceive themselves with more positive traits (Galor and Hentschel, 2013). These strategies work only in the short term. Stereotypes could have the function to protect the professional from negative feelings (Fernández Ballesteros et al., 2017). However, in the long term, these may have negative effects (Casas et al., 2002) for both professionals and the older adults affected. In other studies, they specified that some strategies such as self-efficacy, self-control, techniques for dealing with frustration, or resilience can reduce the effects of burnout (Gutierrez-Martínez et al., 2021).

In spite of the contributions of this research, there are several limitations in this study that it should be mentioned. First, there is a lack of investigation in variables moderating the relationship between burnout and QoL so we cannot compare our results with previous research. Nevertheless, our research explained 46–48% of the variance. Second, the moderations were not highly significant. However, this is the starting point for further research on these variables. Third, it is the size of the sample, as to whether it is truly representative of the population with 312 professionals, although it is a similar sample to that found in other studies (Zamora and Sánchez, 2008; Fernández Ballesteros et al., 2017; Iecovich and Avivi, 2017; Mantzorou et al., 2020; Martínez-López et al., 2021). And finally, as this is a cross-sectional study, it would be important to check what would happen to these variables over time.

Despite these limitations, our results allow us to propose new areas in which intervene for reducing the impact of burnout on professionals QoL. Burnout is frequent in a large extent of nursing home professionals. This research supports the importance of developing interventions focused on beliefs about older people, as they relate to the burnout of nursing home professionals (Mantzorou et al., 2020) which, in turn, negatively affects professionals’ quality of life (Harrad and Sulla, 2018). It is also important to think about protecting professionals in the long term, for example, interventions to improve coping and stress management skills (Martínez-López et al., 2021) can help reduce factors that are associated with burnout, such as emotional demands or support from peers or supervisors (Andela et al., 2021), and even stereotypes toward older people. It is necessary to promote good treatment in care homes that fosters humanization, personalization, respect, and empowerment (Pérez-Rojo et al., 2021).

Over half of the family caregivers indicated that they had some desire to institutionalize their older adults’ relatives (López et al., 2012). Nevertheless, only 3.55% of Spanish older adults live in a nursing home (Abellan et al., 2020). These data will be probably affected by the COVID-19 pandemic. Nursing homes residents and professionals have especially suffered the impact of COVID-19 pandemic. A better professional’s quality of life and less burnout could help to improve residents’ conditions.

## Data Availability Statement

The raw data supporting the conclusions of this article will be made available by the authors, without undue reservation.

## Ethics Statement

The studies involving human participants were reviewed and approved by the University CEU San Pablo Ethics Committee. The patients/participants provided their written informed consent to participate in this study.

## Author Contributions

PL-F, GP-R, JL, and JM-H: study design. PL-F and LG: data collection. PL-F, JM-H, GP-R, and CN: data analysis. PL-F, GP-R, JL, CN, and CV: study supervision. PL-F, GP-R, JM-H, JL, CN, and CV: manuscript writing. PL-F, IC, GP-R, CN, CV, and JL: critical revisions for important intellectual content. All authors contributed to the article and approved the submitted version.

## Conflict of Interest

The authors declare that the research was conducted in the absence of any commercial or financial relationships that could be construed as a potential conflict of interest.

## Publisher’s Note

All claims expressed in this article are solely those of the authors and do not necessarily represent those of their affiliated organizations, or those of the publisher, the editors and the reviewers. Any product that may be evaluated in this article, or claim that may be made by its manufacturer, is not guaranteed or endorsed by the publisher.
